# Supplementary material for: Complexions at the iron-magnetite interface
Source: Nat Commun. 2025 Mar 19;16:2705. doi: 10.1038/s41467-025-58022-y (PMC11923288; doi:10.1038/s41467-025-58022-y)
Supplement: Supplementary file 1 — Supplementary Information [file 41467_2025_58022_MOESM1_ESM.pdf]

Supplementary Information for Complexions at the Iron-Magnetite Interface

Xuyang Zhou[1*], Baptiste Bienvenu[1*], Yuxiang Wu[1], Alisson Kwiatkowski da Silva[1], Colin Ophus[2] and Dierk Raabe[1*]

[1]Max-Planck-Institut for Sustainable Materials (Max-Planck-Institut für Eisenforschung), Max-Planck-Straße 1, Düsseldorf, 40237, Germany.
[2]National Center for Electron Microscopy, The Molecular Foundry, Lawrence Berkeley National Laboratory, Berkeley, 94720, USA.

*Corresponding author(s). E-mail(s): x.zhou@mpie.de; b.bienvenu@mpie.de; raabe@mpie.de;
Contributing authors: yuxiang.wu@mpie.de; a.kwdasilva@mpie.de; clophus@lbl.gov;

This PDF file includes:

Supplementary Table 1 and Table 2

Supplementary Figure 1 to Figure 23

Supplementary References

Supplemental Tables

Supplementary Table 1: Lattice constants ($a_0 = a = b = c$, in Å) and bulk modulus B_0 (in GPa) of cubic BCC-Fe, FeO and Fe_3O_4 obtained using DFT (GGA-PBE) and DFT + U (GGA-PBE with $U_{Fe} = 4\,eV$) compared to experimental data from indicated references.

	DFT		DFT + U_{Fe}		Expt.	
Material	a_0	B_0	a_0	B_0	a_0	B_0
Fe (BCC/FM)	2.83	188	2.95	123	2.87 [1]	164 [2]
FeO (NaCl/AF)	4.30	172	4.34	164	4.30 − 4.33 [3]	150 − 180 [3]
Fe_3O_4 (spinel/FeM)	8.40	172	8.47	191	8.40 [4]	183 [4]

Supplementary Table 2: In-plane lattice mismatch ε_x and ε_y (in %) along the X and Y axis respectively, and adhesion energy γ_{adh} (in J/m^2). All properties are reported for both the initial (upper values) and the fully relaxed geometries, *i.e.* atomic positions and the geometry of the cell (lower values).

	(ε_x, ε_y)			
Interface	Fe	FeO	Fe_3O_4	γ_{adh}
Fe/Fe_3O_4	(+2.4, +2.4)<br>(+2.9, +2.8)	/	(−2.3, −2.3)<br>(−1.8, −1.9)	−2.58<br>−2.96
Fe/FeO	(+3.6, +3.6)<br>(+3.2, −0.5)	(−3.4, −3.4)<br>(−3.8, −7.3)	/	−2.11<br>−3.68
FeO/Fe_3O_4	/	(−1.2, −1.2)<br>(+0.3, −3.5)	(+1.2, +1.2)<br>(+2.8, −1.1)	−1.76<br>−2.49
$Fe/\{FeO\}_2/Fe_3O_4$	(+4.0, +4.0)<br>(+4.7, +2.7)	(−3.1, −3.1)<br>(−2.4, −4.3)	(−0.7, −0.7)<br>(−0.1, −1.9)	−3.74<br>/

Supplemental Figures

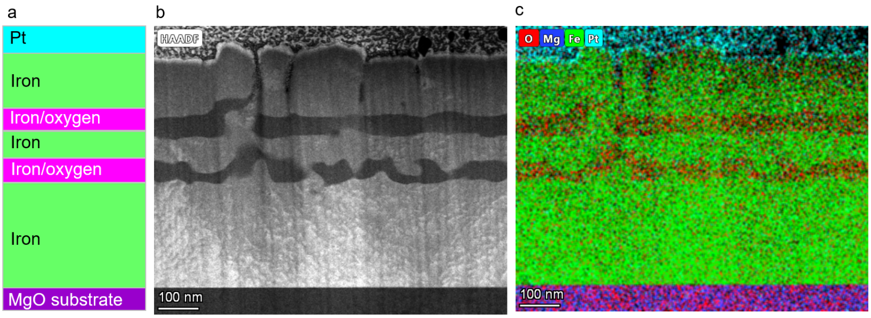

Supplementary Figure 1: Overview of the multi-layer thin film. a An illustrative representation of the structure; **b** STEM - High-angle annular dark field (HAADF) image and **c** EDS map showing the cross-section view of the thin film.

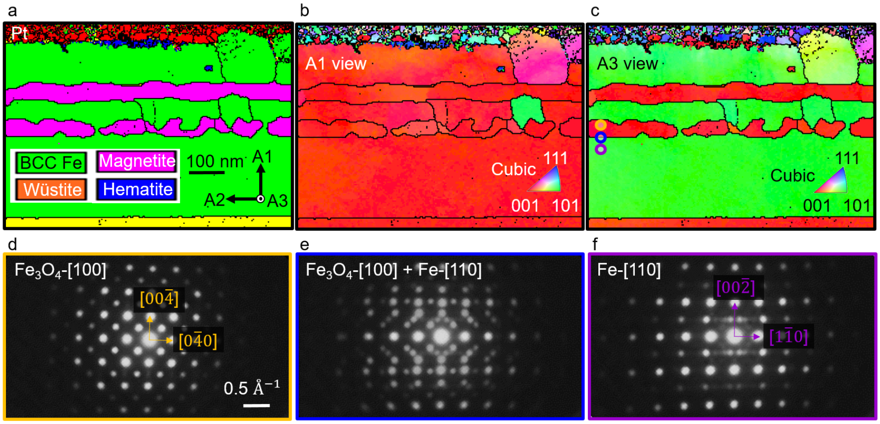

Supplementary Figure 2: Structural characterization of the multi-layer thin film. a Phase map and orientation maps obtained from **b** View A1 and **c** View A3, reconstructed from the precession-assisted 4DSTEM datasets of the cross-sectional view of the thin film. The sample coordinates A1-A3 were defined in the phase map. **d-f** Diffraction patterns from regions highlighted in **c** (orange, blue, and purple circles) are **d** magnetite-[100], **e** the interface between magnetite-[100] and Fe-[110], and **f** Fe-[110]. Additional diffraction spots in **d** and **f**, not part of the Fe or Fe_3O_4 lattice, arise from the native surface oxide layer on Fe.

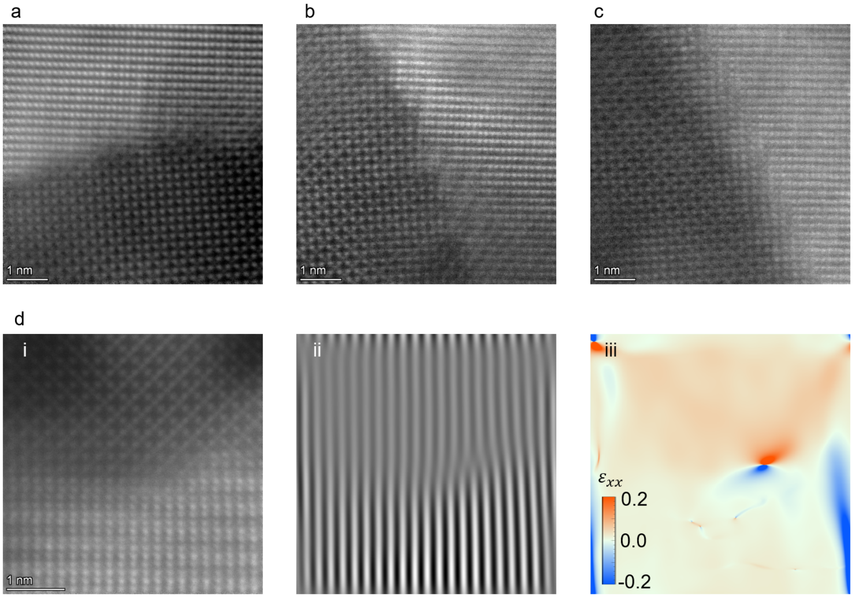

Supplementary Figure 3: Atomic structural characterization of the multi-layer thin film. a-d display high-resolution STEM-HAADF images of the interface between Fe and Fe_3O_4. **d-ii-iii** further present the Bragg-filtered phase map and strain map for the same regions as shown in **d-i**.

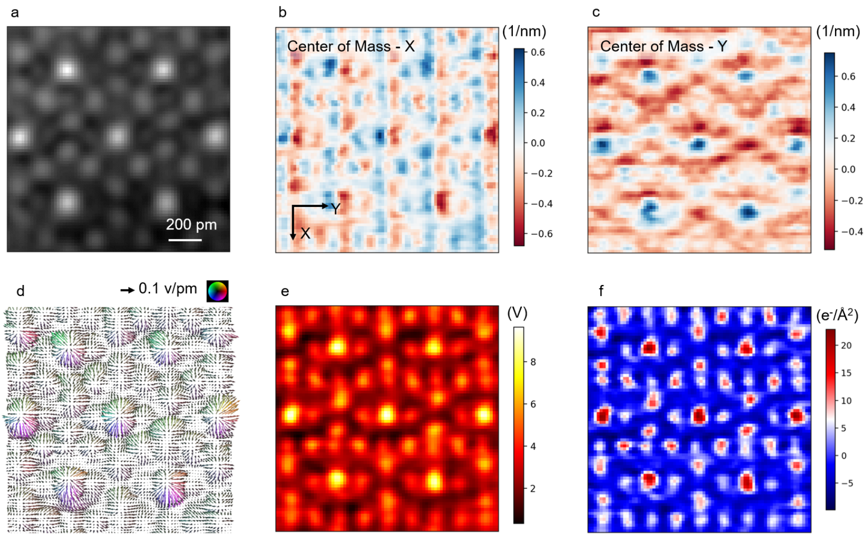

Supplementary Figure 4: Experimental DPC-4DSTEM reconstruction for Fe_3O_4 oriented in the [110] **direction. a** Reconstructed virtual dark-field image. Change of the center of mass of the transmitted beam in **b** X and **c** Y directions. **d** Electric field vector map. **e** Projected electrostatic potential map. **f** Charge-density map. The scanning step size used in this experiment is 13 pm.

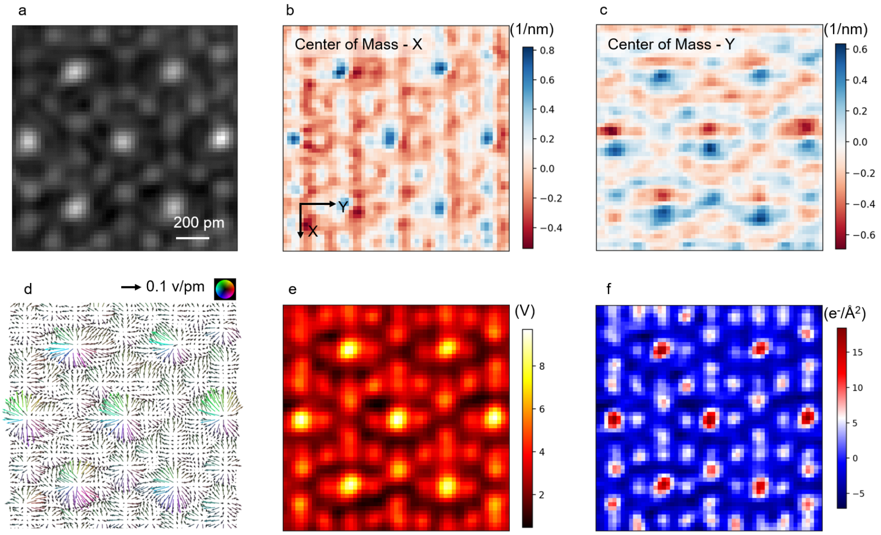

Supplementary Figure 5: Experimental DPC-4DSTEM reconstruction for Fe_3O_4 oriented in the [110] direction. a Reconstructed virtual dark-field image. Change of the center of mass of the transmitted beam in **b** X and **c** Y directions. **d** Electric field vector map. **d** Projected electrostatic potential map. **f** Charge-density map. The scanning step size used in this experiment is 18 pm.

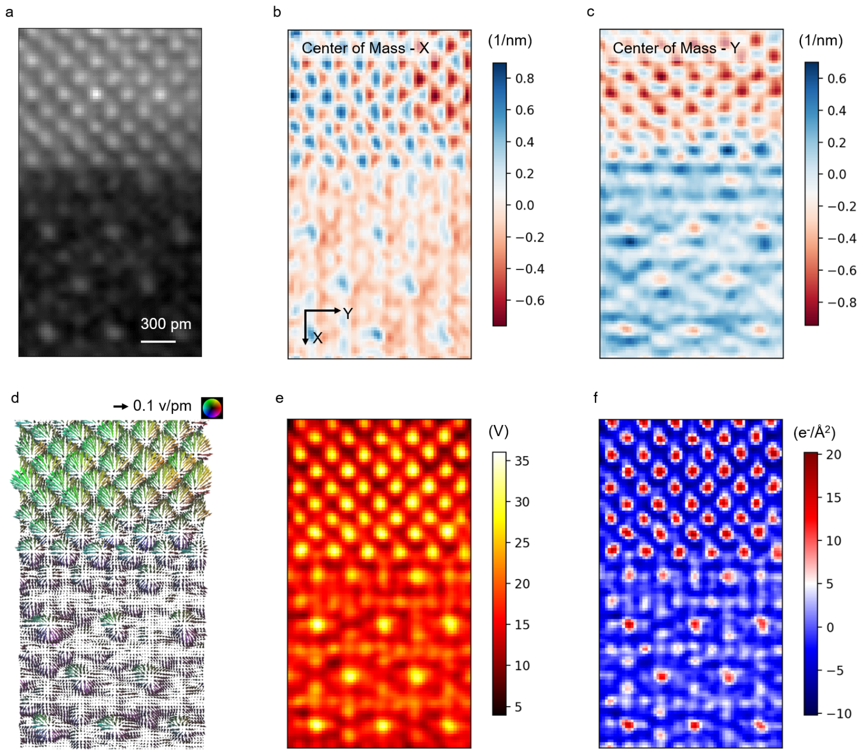

Supplementary Figure 6: Experimental DPC-4DSTEM reconstruction for the Fe/Fe_3O_4 interface with the Fe_3O_4 oriented in the [110] direction. a Reconstructed virtual dark-field image. Change of the center of mass of the transmitted beam in **b** X and **c** Y directions. **d** Electric field vector map. **e** Projected electrostatic potential map. **f** Charge-density map. The scanning step size used in this experiment is 18 pm.

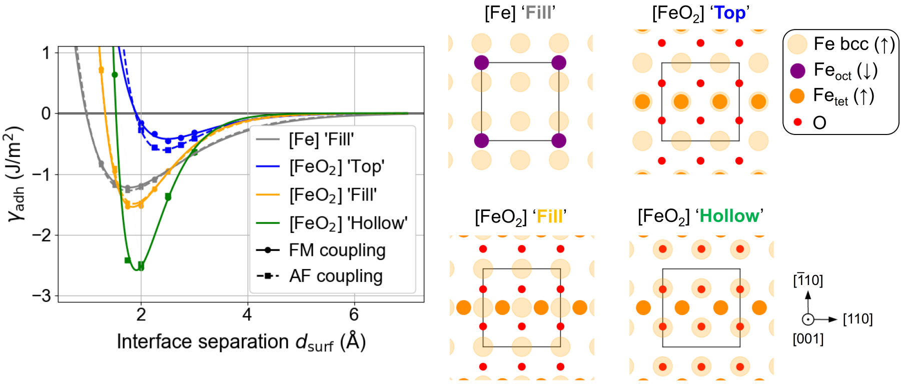

Supplementary Figure 7: Stable sites for the Fe/Fe_3O_4 interface Adhesion energy γ_{adh} of the Fe(001)-Fe_3O_4(001) interface as a function of the interface separation distance d_{surf} for different relative positions of the two layers. The corresponding sites are shown on the right, viewed along the [001] direction of the two structures.

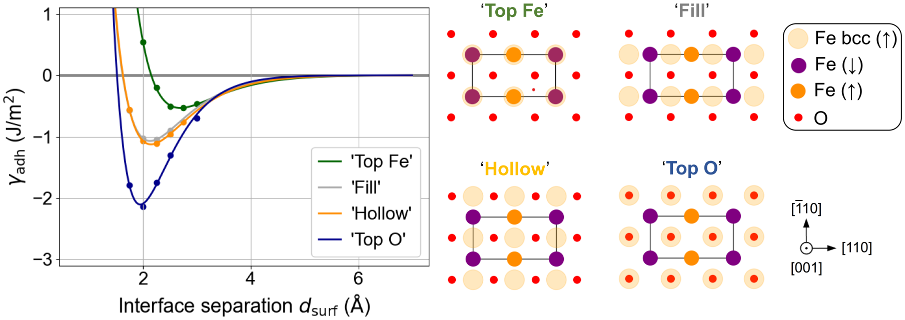

Supplementary Figure 8: Stable sites for the Fe/FeO interface Adhesion energy γ_{adh} of the Fe(001)-FeO(001) interface as a function of the interface separation distance d_{surf} for different relative positions of the two layers. The corresponding sites are shown on the right, viewed along the [001] direction of the two structures.

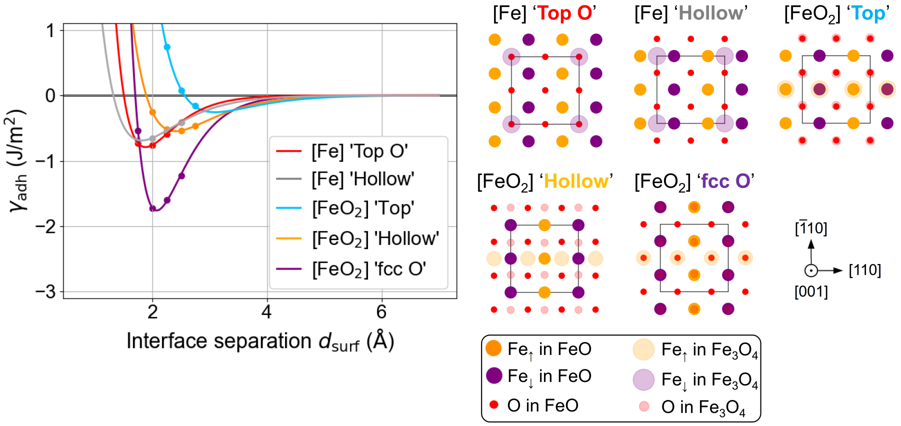

Supplementary Figure 9: Stable sites for the FeO-Fe_3O_4 interface Adhesion energy $\gamma_{\rm adh}$ of the FeO(001)-Fe_3O_4(001) interface as a function of the interface separation distance $d_{\rm surf}$ for different relative positions of the two layers. The corresponding sites are shown on the right, viewed along the [001] direction of the two structures.

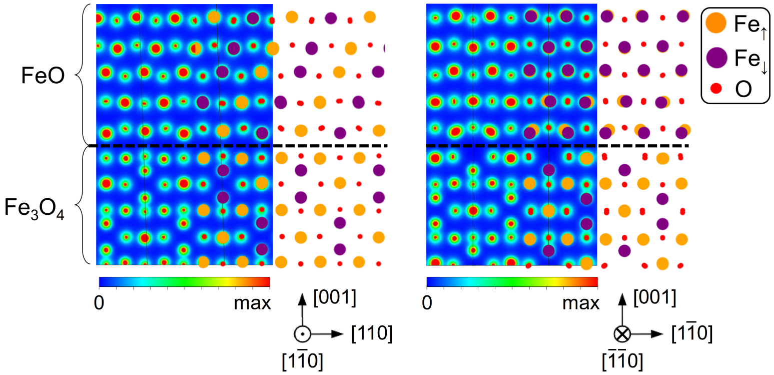

Supplementary Figure 10: The relaxed FeO-Fe_3O_4 interface Charge density and relaxed atomic structure of the FeO(001)-Fe_3O_4(001) interface obtained using DFT calculations. With respect to Supplementary Fig. 9, the configuration of the interface corresponds to the [FeO_2] "fcc O" site, where the two fcc O sub-lattices of FeO and Fe_3O_4 match along their respective [001] direction.

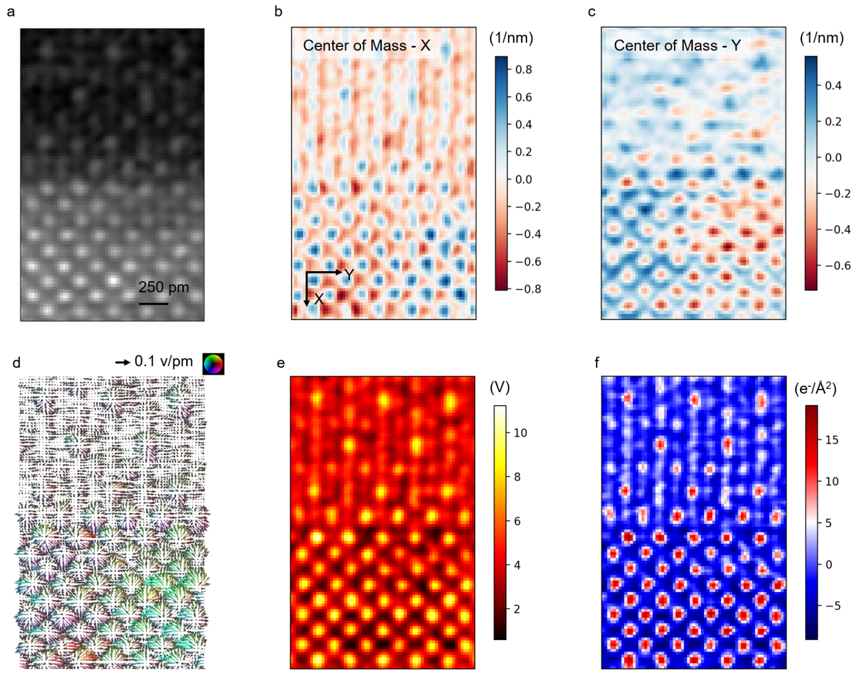

Supplementary Figure 11: Experimental DPC-4DSTEM reconstruction for the Fe_3O_4-Fe interface with the Fe_3O_4 oriented in the [110] direction. **a** Reconstructed virtual dark-field image. Change of the center of mass of the transmitted beam in **b** X and **c** Y directions. **d** Electric field vector map. **e** Projected electrostatic potential map. **f** Charge-density map. The scanning step size used in this experiment is 18 pm.

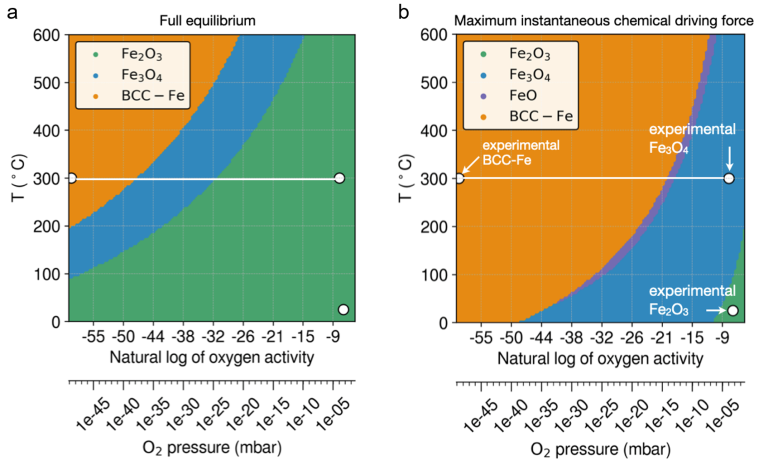

Supplementary Figure 12: Bulk thermodynamics in thin film interface fabrication. a Equilibrium phase diagram as a function of O activity and temperature; **b** The maximum instantaneous chemical driving force diagram illustrates the phases with the maximum instantaneous chemical driving force as a function of O activity and temperature. Three distinct external O activities lead to three possible syntheses of phases. A detailed presentation of the maximum instantaneous chemical driving force mapping is shown in Supplementary Fig. 13.

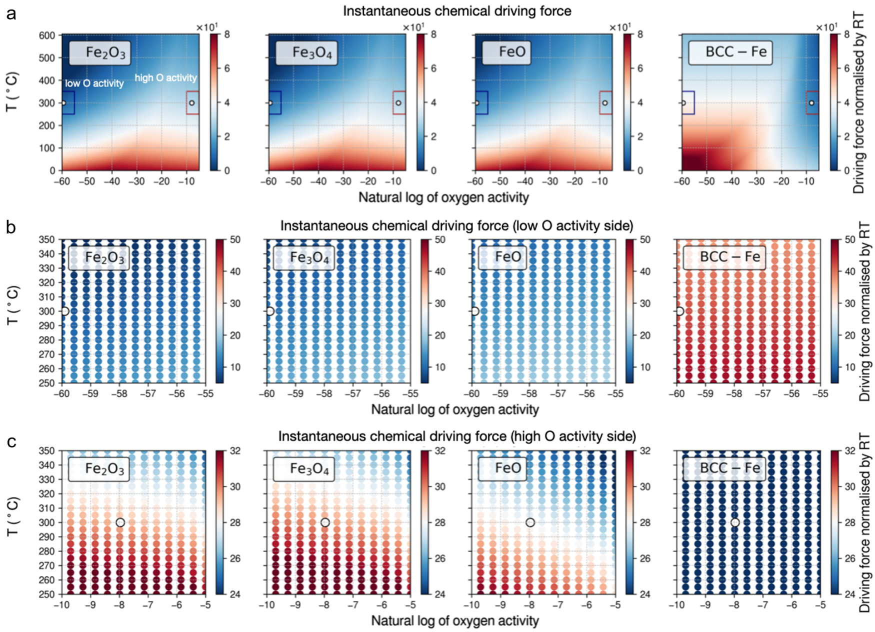

Supplementary Figure 13: The maximum instantaneous chemical driving force as a function of temperature and O activity. a Mapping of the maximum instantaneous chemical driving force for the deposition of solid phases from the gas phase. **b & c** Enlarged view of the maximum instantaneous chemical driving force landscape in the region of low O activity and high O activity regimens respectively, as highlighted in **a**.

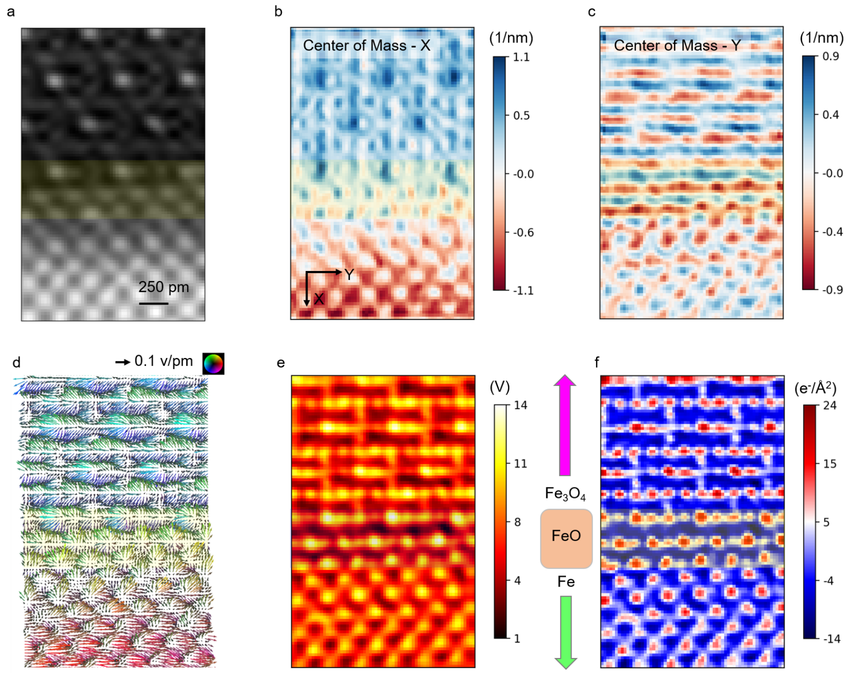

Supplementary Figure 14: Experimental DPC-4DSTEM reconstruction for a three-layer thick FeO-like slab at the Fe/Fe_3O_4 interface, with Fe_3O_4oriented in the [110] direction. **a** Reconstructed virtual dark-field image. Change of the center of mass of the transmitted beam in **b** X and **c** Y directions. **d** Electric field vector map. **e** Projected electrostatic potential map. **f** Charge-density map. The scanning step size used in this experiment is 18 pm. The specimen is the same as shown in Supplementary Fig. 19, which underwent *in situ* heating in TEM at 300 °C for 2 h.

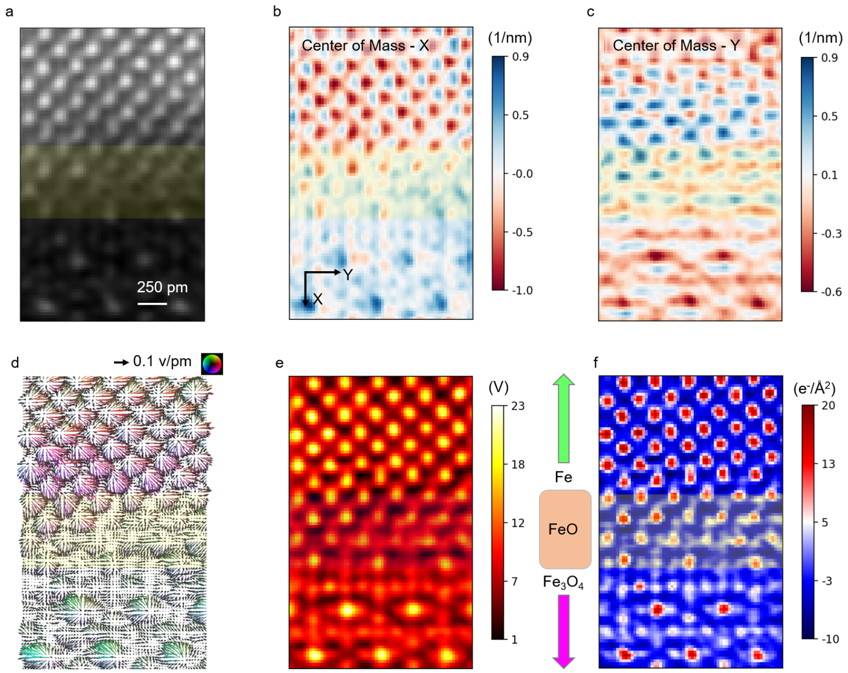

Supplementary Figure 15: Experimental DPC-4DSTEM reconstruction for a four-layer thick FeO-like slab at the Fe/Fe_3O_4 interface, with Fe_3O_4oriented in the [110] direction. a Reconstructed virtual dark-field image. Change of the center of mass of the transmitted beam in **b** X and **c** Y directions. **d** Electric field vector map. **e** Projected electrostatic potential map. **f** Charge-density map. The scanning step size used in this experiment is 18 pm.

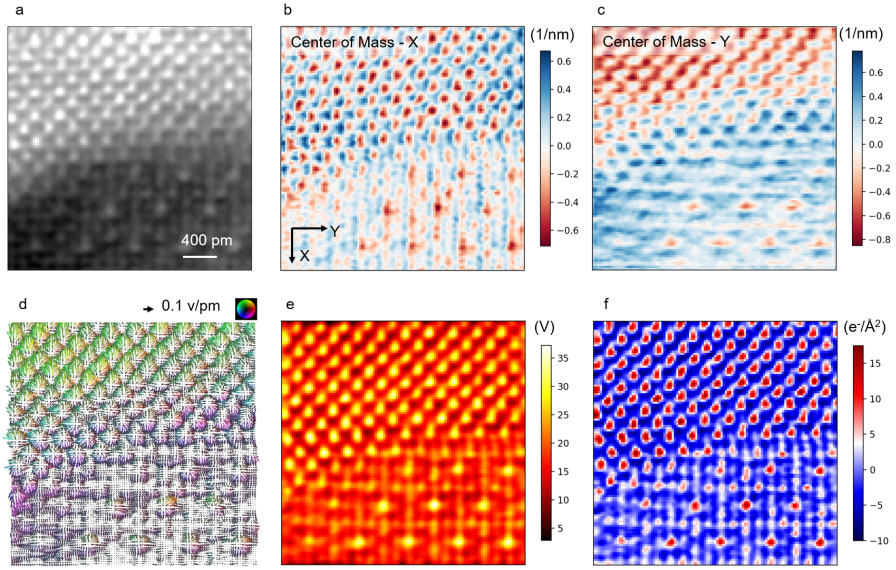

Supplementary Figure 16: Experimental DPC-4DSTEM reconstruction for the Fe/Fe_3O_4 interface with a step, Fe_3O_4 oriented in the [110] direction. **a** Reconstructed virtual dark-field image. Change of the center of mass of the transmitted beam in **b** X and **c** Y directions. **d** Electric field vector map. **e** Projected electrostatic potential map. **f** Charge-density map. The scanning step size used in this experiment is 18 pm.

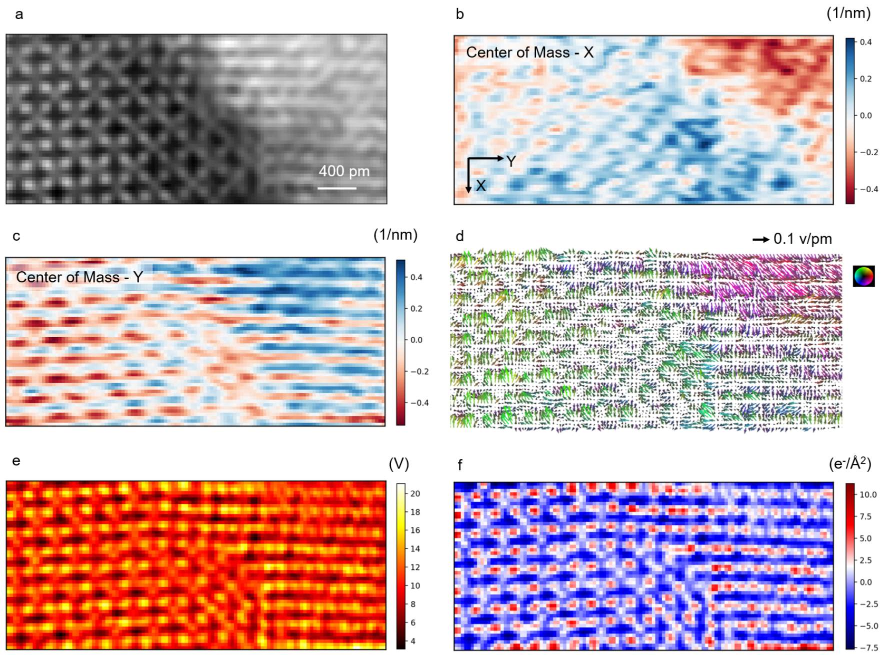

Supplementary Figure 17: Experimental DPC-4DSTEM reconstruction of the semi-coherent interface between Fe and Fe_3O_4, with Fe_3O_4 oriented in the [100] **direction. a** Reconstructed virtual dark-field image. Change of the center of mass of the transmitted beam in **b** X and **c** Y directions. **d** Electric field vector map. **e** Projected electrostatic potential map. **f** Charge-density map. The scanning step size used in this experiment is 25 pm.

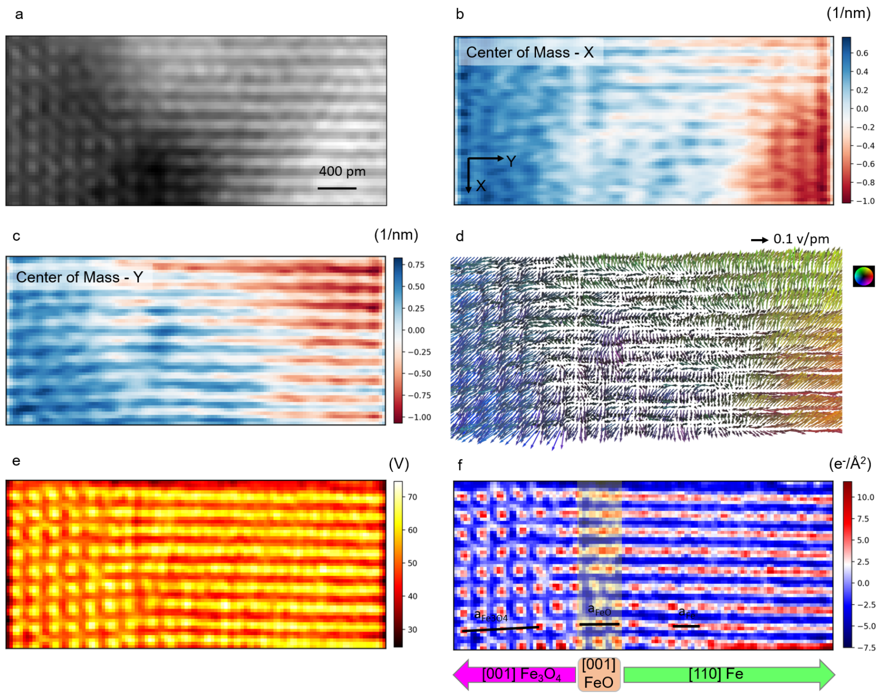

Supplementary Figure 18: Experimental DPC-4DSTEM reconstruction of the coherent interface between Fe and Fe_3O_4, with Fe_3O_4 oriented in the $[100]$ direction. a Reconstructed virtual dark-field image. Change of the center of mass of the transmitted beam in **b** X and **c** Y directions. **d** Electric field vector map. **e** Projected electrostatic potential map. **f** Charge-density map. The scanning step size used in this experiment is 25 pm. Approximate lattice parameters ($a_{Fe_3O_4}$, a_{FeO} and a_{Fe}) are highlighted as black lines between layers of atoms.

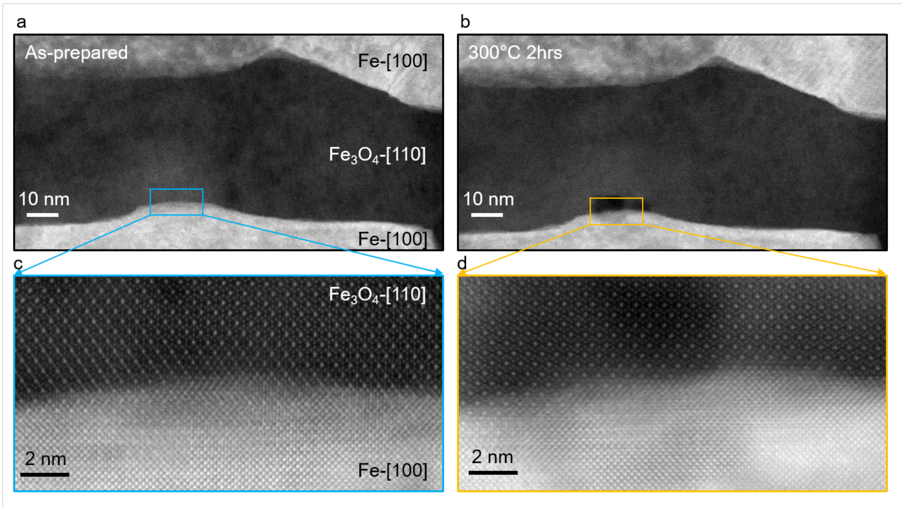

Supplementary Figure 19: Comparison of the interface structure between Fe and Fe_3O_4 before and after *in situ* heating experiments. **a** Overview HAADF imaging of the Fe-Fe_3O_4-Fe structure in the as-prepared condition. **b** The same structure after *in situ* heating in TEM at 300 °C for 2 h, with a heating and cooling rate of 5 °C/s. Both images were taken at room temperature. **c & d** Magnified HAADF images that show the atomic structure of the interface between Fe_3O_4-[110] and Fe-[100].

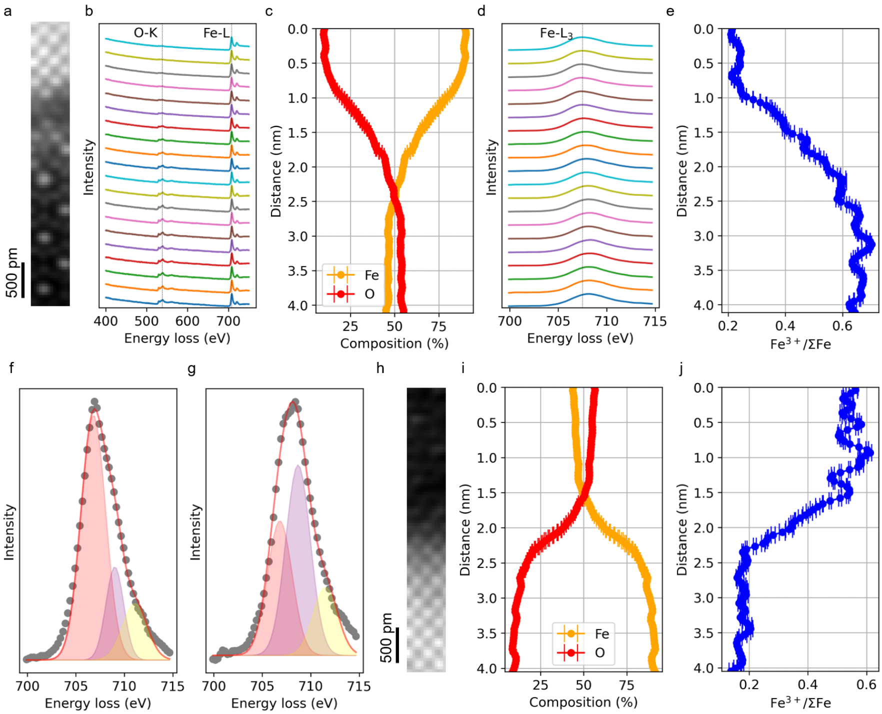

Supplementary Figure 20: Local chemistry and charge state analysis for complexions at the Fe-Fe_3O_4 interface. a Dark-field image serves for electron energy loss spectroscopy (EELS) analysis of the Fe-Fe3O4 interface. **b** Selected energy loss spectra for scanning regions across the Fe-Fe_3O_4 interface correspond to regions in the dark-field image from a. The spectra highlight the O-K edge and Fe-L edges. **c** Quantified local composition using EELS for regions depicted in a, with distances from 0 nm-4.2 nm matching the dark-field image from top to bottom. **d** Magnified regions of the spectra from b show the Fe-L_3 peak, revealing a shift to a higher energy state from the Fe region (upper part) to the Fe_3O_4 (lower part) region. **e** Quantified the charge state using EELS for the regions shown in a. **f & g** display selected spectra for peak decomposition in the Fe region and the Fe_3O_4 region, respectively, using three Gaussian peaks (pink, purple, and yellow) for decomposition. The ratio of $Fe^{3+}/\Sigma Fe$ inversely relates to the integrated area of the pink peak [5]. **h-j** present the dark-field image for the EELS analysis and quantification of local composition and charge state for the scanning regions across the Fe_3O_4-Fe interface.

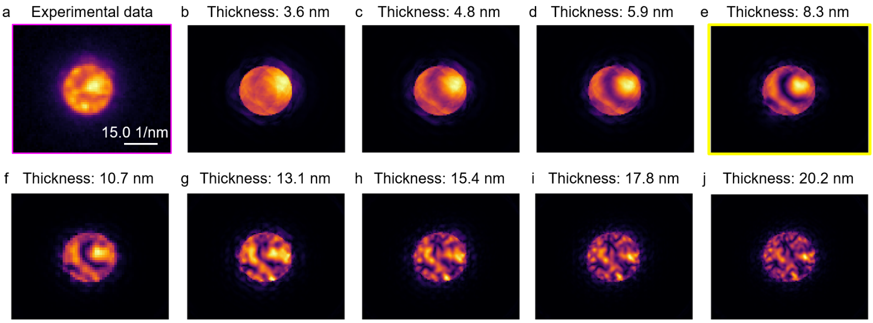

Supplementary Figure 21: Measurement of sample thickness by convergent beam electron diffraction (CBED). a displays an example of an experimental CBED pattern. **b-j** include similar simulated CBED patterns for a range of thicknesses from 3.6 nm to 20.2 nm.

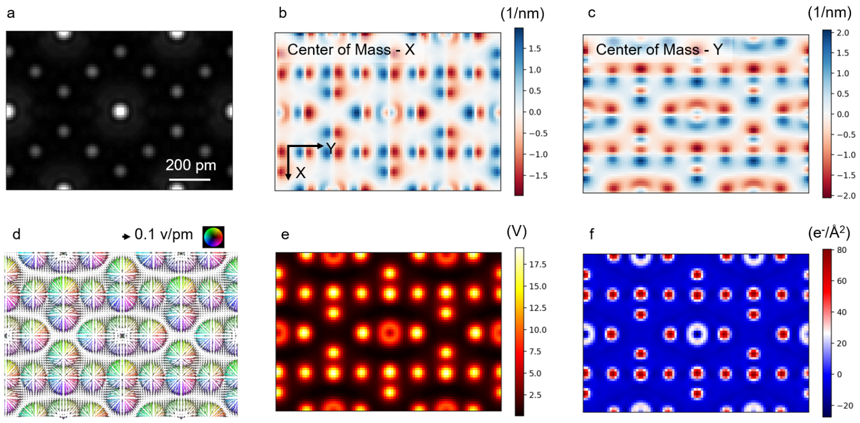

Supplementary Figure 22: Simulated DPC-4DSTEM reconstruction for Fe_3O_4 oriented in the [110] direction. **a** Reconstructed virtual dark-field image. Change of the center of mass of the transmitted beam in **b** X and **c** Y directions. **d** Electric field vector map. **e** Projected electrostatic potential map. **f** Charge-density map. The scanning step size used in this experiment is 12 pm.

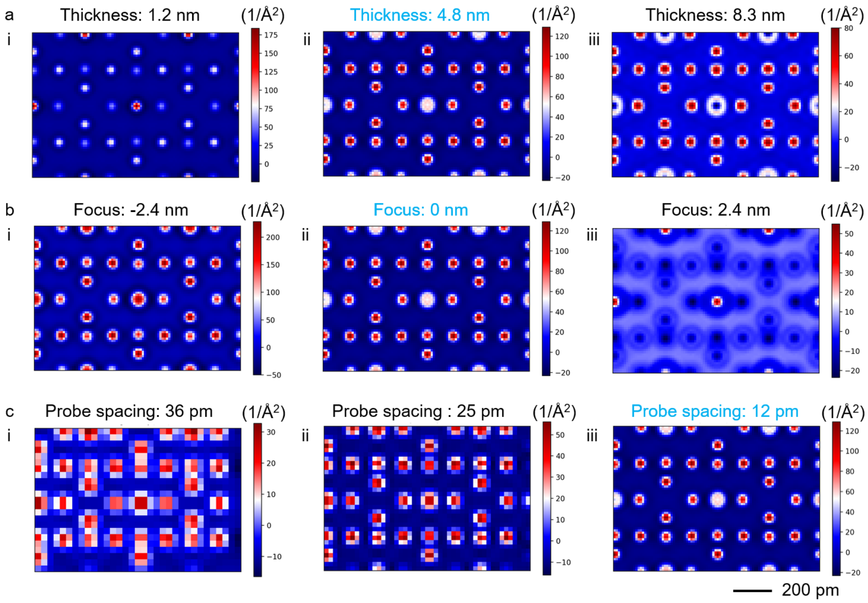

Supplementary Figure 23: The effect of scanning parameters on the reconstructed charge-density maps: **a-i-iii** show the effect of sample thickness, ranging from 1.2 nm to 8.3 nm. **b-i-iii** illustrate the impact of defocus, ranging from -2.4 nm to 2.4 nm. **c-i-iii** demonstrate the influence of probe spacing, ranging from 36 pm to 12 pm. All charge-density maps were reconstructed from simulated DPC-4DSTEM data of the Fe_3O_4 crystal oriented in the (110) direction.

Supplementary References

[1] Kittel, C.: Introduction to Solid State Physics. Wiley, New York (1966)

[2] Dorogokupets, P.I., Dymshits, A.M., Litasov, K.D., Sokolova, T.S.: Thermodynamics and equations of state of iron to 350 GPa and 6000 K. *Sci. Rep.* **7**, 41863 (2017). https://doi.org/10.1038/srep41863

[3] McCammon, C.A., Liu, L.-g.: The effects of pressure and temperature on nonstoichiometric wüstite, Fe_xO: The iron-rich phase boundary. *Phys. Chem. Minerals* **10**, 106–113 (1984). https://doi.org/10.1007/BF00309644

[4] Haavik, C., Stølen, S., Fjellvåg, H., Hanfland, M., Häusermann, D.: Equation of state of magnetite and its high-pressure modification: Thermodynamics of the Fe-O system at high pressure. *American Mineralogist* **85**, 514–523 (2000). https://doi.org/10.2138/am-2000-0413

[5] van Aken, P.A., Liebscher, B.: Quantification of ferrous/ferric ratios in minerals: new evaluation schemes of fe l 23 electron energy-loss near-edge spectra. *Physics and Chemistry of Minerals* **29**, 188–200 (2002). https://doi.org/10.1007/s00269-001-0222-6
